# Feasibility of Recruiting Latinos for a Lung Cancer Screening Study Through a Patient Portal

**DOI:** 10.1111/1759-7714.70067

**Published:** 2025-04-22

**Authors:** Arlette Chávez‐Iñiguez, Chris De Los Reyes, Jeffrey W. Ramos‐Santiago, Rafael H. Orfin, Victoria Uceda, Cody Gardner, Mary Jo Evans, Scott McIntosh, David H. Adler, Ana Paula Cupertino, M. Patricia Rivera, Francisco Cartujano‐Barrera

**Affiliations:** ^1^ Department of Public Health Sciences University of Rochester Medical Center Rochester New York USA; ^2^ Department of Surgery University of Rochester Medical Center Rochester New York USA; ^3^ Clinical and Translational Science Institute University of Rochester Medical Center Rochester New York USA; ^4^ Department of Imaging Sciences University of Rochester Medical Center Rochester New York USA; ^5^ Department of Emergency Medicine University of Rochester Medical Center Rochester New York USA; ^6^ Department of Medicine University of Rochester Medical Center Rochester New York USA

**Keywords:** Latinos, lung cancer, lung cancer screening, patient portal

## Abstract

The purpose of the present study was to assess the feasibility of recruiting Latinos for a lung cancer screening study through a patient portal. The electronic health record (EHR) at the University of Rochester Medical Center (URMC) was utilized to identify individuals with the following characteristics: (1) Hispanic/Latino ethnicity, (2) between 50 and 80 years old, (3) currently smoking, and (4) seen at URMC within the last 10 years. Identified individuals with an active account on MyChart (the patient portal at URMC) were messaged up to two times. The MyChart message, sent in both English and Spanish, invited individuals to participate in a study that aimed to increase the uptake of lung cancer screening among Latinos. A total of 1745 individuals in the EHR at URMC were identified as potentially eligible. Six hundred and sixty‐eight individuals (38.3%, 668/1745) had an active MyChart account and were messaged. Forty‐six individuals responded as interested in the study (the *study interest rate* was 6.8%, 46/668). Ten individuals were eligible and nine enrolled in the study (the *overall enrollment rate* was 1.3%, 9/668, and the *enrollment/eligible rate* was 90%, 9/10). It is feasible to recruit Latinos for a lung cancer screening study through a patient portal. The study interest, overall enrollment, and enrollment/eligible rates are promising and have the potential to inform future recruitment efforts because of their high‐reach and low‐cost.

## Graphical abstract

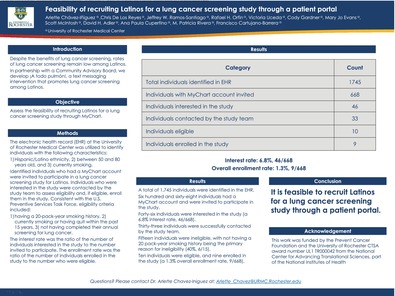

## 1 Introduction

Lung cancer screening decreases lung cancer mortality by early detection [1, 2]. Despite the benefits of lung cancer screening, rates of lung cancer screening remain low among Latinos [3], the largest minority group in the U.S. [4] In response, research efforts to increase the uptake of lung cancer screening among Latinos are emerging [5, 6, 7, 8]. The purpose of the present study was to assess the feasibility of recruiting Latinos for a lung cancer screening study through a patient portal.

## 2 Methods

This is a secondary data analysis of the recruitment for the *A todo pulmón* study. *A todo pulmón* is a single‐arm pilot study assessing the feasibility, acceptability, and preliminary impact of a culturally appropriate text messaging intervention to increase the uptake of lung cancer screening among Latinos. A community advisory board of Latino stakeholders guided the *A todo pulmón* study implementation. Study procedures were approved and monitored by the University of Rochester Medical Center (URMC) Institutional Review Board (protocol number STUDY00009079).

In June 2024, the electronic health record (EHR) at URMC was utilized to identify individuals with the following characteristics: (1) Hispanic/Latino ethnicity, (2) between 50 and 80 years old, (3) currently smoking, and (4) seen at URMC within the last 10 years. Between July and September 2024, identified individuals with an active account on MyChart (the patient portal at URMC) were messaged up to two times. The MyChart message, sent in both English and Spanish, said: “We are testing a text messaging program to promote lung cancer screening among Latinos. If you enroll, you will receive one text message per week for 12 weeks to learn more about lung cancer screening. Participants can earn up to $80 for completing all study assessments. If you are interested, a member of the study team will contact you to confirm eligibility and to discuss the next steps to participate.”

The study team made five attempts to contact individuals who responded as interested in the study through phone calls to assess eligibility. Consistent with the U.S. Preventive Services Task Force (USPSTF), eligibility criteria included: (1) having a 20‐pack‐year smoking history, and (2) currently smoking or having quit within the past 15 years [9]. Moreover, eligibility criteria included not having completed their annual screening for lung cancer. Eligible individuals were scheduled for a Zoom or phone call appointment. During the appointment, research staff guided individuals through the process of written informed consent. All study procedures were available in English and Spanish.

*Feasibility* was measured by the study interest, overall enrollment, and enrollment/eligible rates. The *study interest rate* was the ratio of the number of individuals who were interested in the study to the number who were invited to participate. The *overall enrollment rate* was the ratio of the number of individuals enrolled in the study to the number who were invited to participate. The *enrollment/eligible rate* was the ratio of the number of individuals enrolled in the study to the number who were eligible.

## 3 Results

A total of 1745 individuals in the EHR at URMC were identified as potentially eligible. Six hundred and sixty‐eight individuals (38.3%, 668/1745) had an active MyChart account and were invited to participate in the study. Forty‐six individuals responded as interested in the study, and 33 were contacted by the study team. The study team was not able to contact 13 participants. The *study interest rate* was 6.8% (46/668).

Of the 33 individuals who were contacted by the study team, eight did not complete the eligibility assessment (24.2%, 8/33). Fifteen individuals were ineligible (45.5%, 15/33). Not having a 20‐pack‐year smoking history was the primary reason for ineligibility (40%, 6/15). Ten individuals were eligible, and nine enrolled in the study. The *overall enrollment rate* was 1.3% (9/668) and the *enrollment/eligible rate* was 90% (9/10).

## 4 Discussion

To our knowledge, this is the first study assessing the feasibility of recruiting Latinos for a lung cancer screening study through a patient portal. In 2024, Dykes et al. [10] reported that 67% of all URMC patients had an active MyChart account. In our study, only 38.3% of identified individuals had an active MyChart account. This difference may be attributed to two previously described characteristics associated with not having an active account in a patient portal: older age and Latino ethnicity [11]. Regardless of this important difference, this work demonstrates that it is feasible to recruit Latinos for a lung cancer screening study through a patient portal.

Dykes et al. [10] also reported that across 19 different studies (from dermatology to psychiatry studies) at URMC, the *study interest rate* was 7.7% (636/8240). Our *study interest rate* was relatively similar (6.8%). Furthermore, although direct comparisons have not been reported, our *overall enrollment* and *enrollment/eligible* rates (1.3% and 90%, respectively) are promising and have the potential to inform future recruitment efforts because of their high‐reach and low‐cost. This is particularly relevant as Latinos are underrepresented in interventional studies aiming to increase the uptake of lung cancer screening [12].

To address racial and ethnic disparities in lung cancer screening eligibility, the USPSTF updated its guidelines in 2021, lowering the starting age from 55 to 50 and reducing the smoking history criteria from 30 to 20 pack‐years [9]. Despite the updated guidelines, Latinos are less likely to be eligible for lung cancer screening compared to White individuals [13]. In our study, not meeting the 20 pack‐year smoking history requirement was the primary reason for ineligibility. This result is not surprising as light (less than 10 cigarettes per day) and non‐daily smoking patterns are common among Latinos [14].

Some limitations should be considered when interpreting the findings. First, this study was not designed to test the efficacy of recruitment through MyChart. Second, this study had a modest sample size. Third, the demographic information of participants was limited to that included in the EHR. Fourth, this study was solely implemented at URMC. It is possible that deploying this recruitment method in a different setting (e.g., a safety net clinic) may result in varying outcomes. Lastly, individuals were only invited to participate in the study up to two times due to URMC policy. Additional MyChart messages may potentially increase enrollment over time.

## 5 Conclusion

It is feasible to recruit Latinos for a lung cancer screening study through a patient portal.

## Author Contributions

Conceptualization: A.C.‐I., C.G., A.P.C., M.P.R., and F.C.‐B. Data curation: A.C.‐I., C.D.L.R., J.W.R.‐S., and R.H.O. Formal analysis: A.C.‐I. and F.C.‐B. Funding acquisition: A.P.C., M.P.R., and F.C.‐B. Investigation: V.U. Project administration: R.H.O. Writing – original draft preparation: A.C.‐I. and F.C.‐B. Writing – review and editing: C.D.L.R., J.W.R.‐S., R.H.O., V.U., C.G., M.J.E., S.M., D.H.A., A.P.C., and M.P.R. All the authors read and approved the final manuscript.

## Ethics Statement

Study procedures were approved and monitored by the University of Rochester Medical Center (URMC) Institutional Review Board (protocol number STUDY00009079).

## Conflicts of Interest

The authors declare no conflicts of interest.

## Data Availability

The datasets generated for this study are available on request to the corresponding author.
